# Exposure to Cumulus Cell Secretome Improves Sperm Function: New Perspectives for Sperm Selection In Vitro

**DOI:** 10.3390/cells12192349

**Published:** 2023-09-25

**Authors:** Francesca Paola Luongo, Silvia Perez Casasus, Alesandro Haxhiu, Fabio Barbarulo, Marta Scarcella, Laura Governini, Paola Piomboni, Catello Scarica, Alice Luddi

**Affiliations:** 1 Department of Molecular and Developmental Medicine, University of Siena, 53100 Siena, Italy; s.perezcasasus@student.unisi.it (S.P.C.); alesandro.haxhiu@student.unisi.it (A.H.); luongofrancescapaola@gmail.com (F.P.L.); laura.governini@unisi.it (L.G.); alice.luddi@unisi.it (A.L.); 2 New Fertility Group—European Hospital, Centre for Reproductive Medicine (NFG), 00148 Rome, Italym.scarcella@student.unisi.it (M.S.)

**Keywords:** sperm selection, oxygen consumption, cumulus cells, capacitation, tyrosine phosphorylation

## Abstract

In the literature, there is a well-known correlation between poor semen quality and DNA sperm integrity, which can turn into negative outcomes in terms of embryo development and clinical pregnancy. Sperm selection plays a pivotal role in clinical practice, and the most widely used methods are mainly based on sperm motility and morphology. The cumulus oophorus complex (COC) during natural fertilization represents a barrier that spermatozoa must overcome to reach the zona pellucida and fertilize the oocyte. Spermatozoa that can pass through the COC have better structural and metabolic characteristics as well as enhanced acrosome reaction (AR). The present study aimed to evaluate the exposure of sperm to cumulus cell secretome during swim-up treatment (SUC) compared with the routinely used swim-up method (SU). To determine the effectiveness of this method, biological factors critical for the ability of sperm to fertilize an oocyte, including capacitation, AR, tyrosine phosphorylation signature, DNA integrity, and mitochondrial functionality, were assessed. The SUC selection assures recovery of high-quality spermatozoa, with enhanced mitochondrial functionality and motility compared with both SU-selected and unselected (U) sperm. Furthermore, using this modified swim-up procedure, significantly reduced sperm DNA damage (*p* < 0.05) was detected. In conclusion, the SUC approach is a more physiological and integrated method for sperm selection that deserves further investigation for its translation into clinical practice.

## 1. Introduction

Mammalian fertilization involves a series of coordinated cellular and molecular events that ultimately lead to the fusion of the sperm and egg [1,2,3].

During a natural fertilization process, sperm must pass through the cumulus cell layer, consisting of several thousand cells, tightly glued together by the extracellular matrix (ECM) that surrounds the oocyte. There is a significant amount of literature that confirms the importance of both cumulus cells (CCs) and the ECM in the fertilization process. First of all, the sperm head of mature spermatozoa has a hyaluronan-specific receptor that allows them to bind with hyaluronan; studies in mice demonstrated that they cannot penetrate the oocyte without the presence of CCs [4]. In addition, the interaction between CCs and male gametes results in physiological changes in the sperm, which ultimately make them capable of fertilizing [5,6,7]. CCs secrete progesterone, which is a crucial hormone that acts as a chemoattractant and stimulates sperm hyperactivated flagellar movement and acrosome reaction (AR). It has been found that even small concentrations of progesterone can effectively stimulate calcium ion channels localized in the human sperm flagellum [8]. Notably, the processes of sperm motility hyperactivation and AR both involve calcium mobilization.

Moreover, it was reported that progesterone speeds up the process of tyrosine phosphorylation, which is a significant sign of sperm capacitation in sperm, determining the preparation for the AR event [9]. Additionally, numerous studies have demonstrated that CCs provide factors able to impact sperm functions, such as prostaglandins (PGE1, PGE2, and PFG2a). These latter were detected in the incubation medium of cumulus oophorus complexes (COCs); when indomethacin was used to block their biosynthesis, the fertilization rate decreased [10]. Therefore, all these pieces of evidence have brought to light the communication between sperm and the CCs and matrix. These discoveries not only prompt a rethinking of the role of CCs in sperm penetration and fertilization but also open the possibility of their involvement in sperm selection for in vitro fertilization.

Several studies have explored the efficacy of COCs as a more natural approach to select sperm for intracytoplasmic sperm injection (ICSI). In a study conducted by Wang and colleagues [11], oocytes injected with COC-selected spermatozoa showed a higher rate of fertilization compared with the conventional ICSI (85.31% vs. 74.77%). Another study found that spermatozoa successfully penetrating through the cumulus oophorus exhibited higher rates of normal morphology and of AR and had improved motility patterns [12]. Interestingly, hyaluronic acid was found to regulate sperm motility without any impact on AR; on the other hand, CC extract did not affect sperm motility, but it did induce AR. As far as we know, it seems that only one study has been conducted to examine the impact of cumulous cells in sperm selection, in relation to DNA fragmentation. The authors demonstrated a significant decrease in the level of sperm DNA fragmentation in the COC-selected sperm compared with the control [13]. Therefore, sperm selection with CCs for ICSI seems to be effective, even in terms of blastocyst development and quality [11]. However, very little is known about how cumulus cells play a direct and critical role in fertilization and whether the CC components and secreted molecules/vesicles can promote fertilization.

On this basis, this study was designed to investigate the efficacy of sperm exposure to cumulus cell secretome during swim-up treatment compared with the routinely used swim-up method. The effectiveness of this method was assessed by examining biological factors that are critical for the ability of sperm to fertilize an oocyte, including AR, tyrosine phosphorylation signature, DNA integrity, and mitochondrial functionality.

## 2. Materials and Methods

**Scheme 1:**
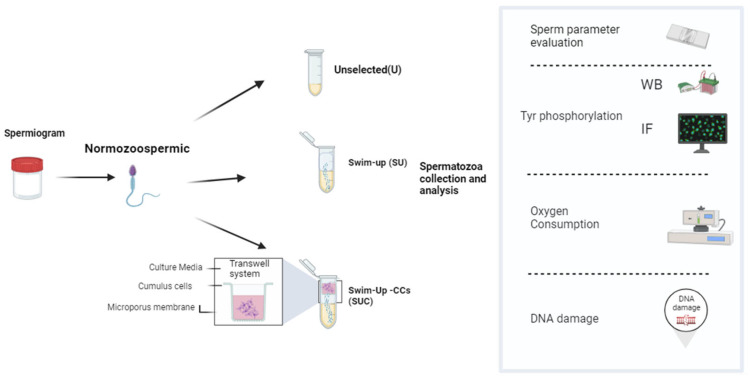
The flowchart of this study.

The flowchart of this study is summarized in the Scheme 1.

### 2.1. Collection of Semen Samples and Semen Analysis

This study was conducted on a cohort of 30 males (mean age: 34.2 years, 16–51) undergoing semen analysis for fertility evaluation at the Unit of Medically Assisted Reproduction, Siena University Hospital, and at the New Fertility Group—European Hospital (NFG) in Rome. The study protocol received approval from the Ethical Committee of the Siena University Hospital (approval ID: CEAVSE, protocol number: 18370, 2 October 2020); before participating, all subjects gave their written informed consent. A comprehensive clinical history was obtained for all participants, and subjects with possible preexisting causes of male infertility, such as varicocele, cryptorchidism, or endocrine disorders, were excluded. The sperm samples were collected via masturbation into sterile containers after a period of abstinence of 2–5 days. After complete liquefaction at room temperature, semen analysis was performed according to World Health Organization criteria [14]. Briefly, 10 μL of well-mixed semen was transferred to a Makler chamber, and the cover was promptly applied, taking care to avoid the formation of bubbles. Seminal parameters (sperm concentration, motility, and morphology) were evaluated by a blinded observer and independently repeated by another blinded observer to ensure quality control. The data reported are the mean value of the two observations. For this study, only normozoospermic samples were included.

### 2.2. COC Collection and Cumulus Cell Culture

After follicular aspiration, COCs were retrieved from follicular fluid and oocytes were isolated from patients attending the Unit of Medically Assisted Reproduction, Siena University Hospital and at the New Fertility Group—European Hospital (NFG) in Rome for ICSI treatment. Women enrolled in this study (*n* = 15; years: < 35) were part of a couple with a diagnosis of male infertility factor. They had regular menstrual cycles and an absence of hormonal disorders, chromosomal abnormalities, and endometrial disease. The ovarian hyperstimulation was induced as previously described [15].

CCs were isolated as previously reported [16] and then cultured at 37 °C with 5% CO_2_ in DMEM supplemented with 10% fetal bovine serum, 1% L-glutamine, 1% penicillin/streptomycin, and 1% non-essential amino acids in a transwell chamber (size: 6.5 mm, pore: 0.4 µm, Corning, Corning, NY, USA).

### 2.3. Sperm Selection with Swim-Up (SU) and Swim-Up-CC (SUC)

For this study, each ejaculate was divided into three aliquots: two aliquots underwent sperm capacitation through either a swim-up process (referred to as SU) or a modified procedure that involved both swim-up and CC interaction (referred to as SUC); one aliquot was evaluated in basal conditions (referred to as unselected, U). To perform SU selection, 1 mL of sperm washing medium (FUJIFILM Irvine Scientific, Santa Ana, CA, USA) was gently layered on an equal volume of the semen sample. After 45 min of incubation at 37 °C, 400 μL of the upper layer of the sample, containing selected sperm, was carefully collected.

In the case of the SUC, the transwell chamber with the CCs was placed on the top of the sperm washing medium. Proteins and soluble or incapsulated factors secreted by CCs can pass through the microporous filter membrane into the lower chamber, while the sperm are retained in the tube by the filter membrane. As in the SU procedure, at the end of the incubation 400 μL of the medium was carefully aspirated from the upper layer of the sample. Sperm analysis according to WHO 2021 was repeated by using 10 µL of each selected sample, while the remaining sperm-selected sample was stored for the subsequent experiments.

### 2.4. Acrosomal Staining

The acrosomal status was determined by using the acrosome marker fluorescein isothiocyanate-labeled Pisum sativum agglutinin (FITC-PSA). Briefly, sperm samples (SUC, SU, and U) were incubated with 30 µg/mL FITC–PSA in PBS for 30 min and washed with distilled water for 10 min. Sperm nuclei were then counterstained with DAPI (6-diamino-2phenylindole) for 10 min at RT. After washing in PBS, the sperm were smeared on glass slides and mounted with DABCO mounting solution. Fluorescence images were acquired by the Leica AF6500 Integrated System for Imaging and Analysis (Leica Microsystems, Wetzlar, Germany), equipped with the LAS software (https://imagej.nih.gov/ij/download.html, accessed on 23 September 2023). Then, the fluorescence intensity was measured with Image-J software (U. S. National Institutes of Health, Bethesda, MD, USA). The percentages of acrosome-reacted sperm/sperm with intact acrosome were determined.

### 2.5. Western Blotting

For Western blot analysis, sperm samples SUC, SU, and U were washed with ice-cold PBS and then lysed using RIPA buffer supplemented with Protease Inhibitor (1:100) and phenylmethylsulphonyl fluoride (PMSF; 1:200). The samples were centrifuged at 12,000× *g* for 20 min at 4 °C, and the soluble protein-containing supernatant was carefully collected for further analysis. Protein concentration was determined by BSA Bradford assay. Western blotting was carried out according to an in-house-developed protocol [17]. From each sample, 25 µg of protein was separated by electrophoresis and then transferred onto nitrocellulose membrane. After blocking, the membrane was incubated overnight at 4 °C with primary antibodies (see Supplementary Table S1), then washed 3 times, and finally incubated with the appropriate HRP-conjugated secondary antibody (see Supplementary Table S1) for 1 h at room temperature. After three washes in PBS-Tween 20 0.1%, immunostained bands were visualized by chemiluminescence with ImageQuant LAS 4000 (GE Healthcare, Chicago, IL, USA). 

### 2.6. Immunofluorescence Staining

Immunofluorescence analysis was carried out following a previously published protocol [18]. Briefly, 50 µL of each sperm sample was washed with PBS and fixed for 15 min in 4% PFA. Subsequently, the sperm were permeabilized with 0.5% Triton X-100 in PBS for 5 min. After blocking in 5% bovine serum albumin (BSA)/1% normal goat serum (NGS) in PBS for 30 min, spermatozoa were incubated with the primary antibody diluted in 1% NGS and 1% BSA in PBS overnight at 4 °C. After three washes with PBS, the cells were then incubated for 1 h at RT with the secondary antibody (Supplementary Table S1). The nuclei were counterstained with DAPI and mounted with DABCO antifade solution. The sperm samples were examined using a Leica AF6500 Integrated System for Imaging and Analysis (Leica Microsystems, Germany) equipped with the LAS software. Staining patterns were analyzed and classified as reported in the literature [19].

### 2.7. Mitochondrial Membrane Potential Assay

MitoTracker staining was used to assess the mitochondrial membrane potential (MMP) of sperm in the three experimental groups, according to a validated protocol [18]. 4′,6-diamidino-2-phenylindole (DAPI) was used to counterstain the nuclei. Sperm were categorized according to the staining of the midpiece. For each sample, at least 200 sperm in at least ten different fields were observed.

### 2.8. Hypotonic Swelling and Oxygraphic Assay

The sperm samples SUC, SU, and U were centrifuged at 800× *g* for 10 min, and the pellet was resuspended in an isotonic salt medium and subjected to hypotonic treatment according to the published protocol [20]. Briefly, sperm cells were incubated in an ice-chilled hypotonic medium consisting of 2 mmol/L of KH2PO4 and 8 mmol/L of K2HPO4 supplemented with 2 g/L of bovine serum albumin (BSA) adjusted to pH 7.4 for 1.5 h. Following hypotonic treatment, sperm were washed three times using the isotonic medium, and the sperm concentration was adjusted to approximately 1–4 million for all samples in each oxygraphic experiment. The measurement of oxygen uptake by spermatozoa was performed at 36 °C using a Clark-type oxygen probe (Hansatech Oxygraph; Pentney, King’s Lynn, UK). Demembranated sperm cells were vigorously stirred in a reaction chamber containing an isotonic salt medium without EDTA and equilibrated to the experimental temperature. The rate of oxygen uptake by the spermatozoa was quantified as nmol O_2_·mL^−1^·min^−1^. The oxygen consumed by the sperm cells was determined by calculating the difference between the initial oxygen level at the time of spermatozoa addition and the oxygen level after 1 min.

### 2.9. Assessment of Sperm DNA Fragmentation

Sperm DNA fragmentation was assessed using the sperm chromatin dispersion test (SCD test; Halosperm G2^®^ assay, Halotech DNA SL, Madrid, Spain). In brief, samples were mixed with an agarose gel before being applied to a precoated slide, refrigerated, and then exposed to a denaturing agent and lysis solution. Slides were then stained and assessed by counting 300 sperm per sample to determine the variability in DNA fragmentation levels (DFLs) [21].

### 2.10. Statistical Analysis

Statistical analysis was performed using the GraphPad Prism 5.0 (GraphPad Software, San Diego, CA, USA). All variables were checked for normal distribution using the one-sample Kolmogorov–Smirnov test. One-way ANOVA was used to compare acrosome staining, oxygen consumption, and DNA fragmentation in the three groups of samples, and post hoc analyses were performed using Tukey’s test. Independent samples *t*-test was used to compare the different sperm subpopulations (MitoTracker positive versus MitoTracker negative) for all numerical variables. Pearson Chi-square was used to compare the results of sperm mitochondrial staining. *p* < 0.05 was considered significant.

## 3. Results

### 3.1. Sperm Parameters in Unselected, SU-Selected, and SUC-Selected Sperm

The baseline characteristics of the semen samples evaluated in this study are summarized in Supplementary Table S2. 

The mean sperm concentration was 75 × 10^6^/mL (range: 20–240). The mean percentage of sperm motility was 65% (47–68), and the normal morphology was 9% (4–16).

In order to assess whether and how CCs may impact the quality of isolated sperm, each semen sample was separated into three aliquots and processed as follow: (1) unselected (U), (2) swim-up only (SU), and (3) swim-up combined with exposure to CC secretome (SUC).

**Figure 1:**
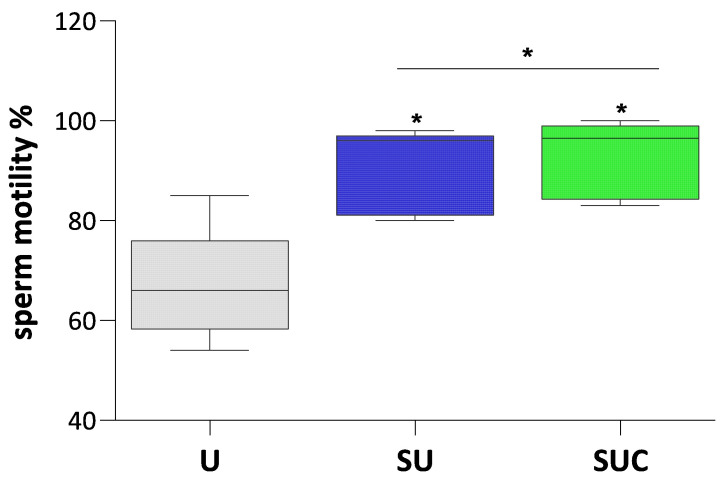
Sperm motility percentage according to WHO 2021 criteria in unselected sperm (U), sperm selected through swim-up (SU), and swim-up combined with exposure to CC secretome (SUC). Graphical diagrams are plotted as box–whisker plots, where boxes show the interquartile range with median and mean values, and whiskers represent min and max confidence intervals. * *p* < 0.05.

The number of sperm collected by SU and SUC was comparable (*p* > 0.05). The effects of the type of selection on sperm motility are shown in Figure 1. Both procedures allow isolating sperm with higher motility, but the sperm collected by the SUC method exhibited a significantly higher motility percentage when compared with those isolated by the SU protocol (*p* < 0.05). 

### 3.2. Acrosomal Reaction Rate and Tyrosine Phosphorylation in Selected Sperm

**Figure 2:**
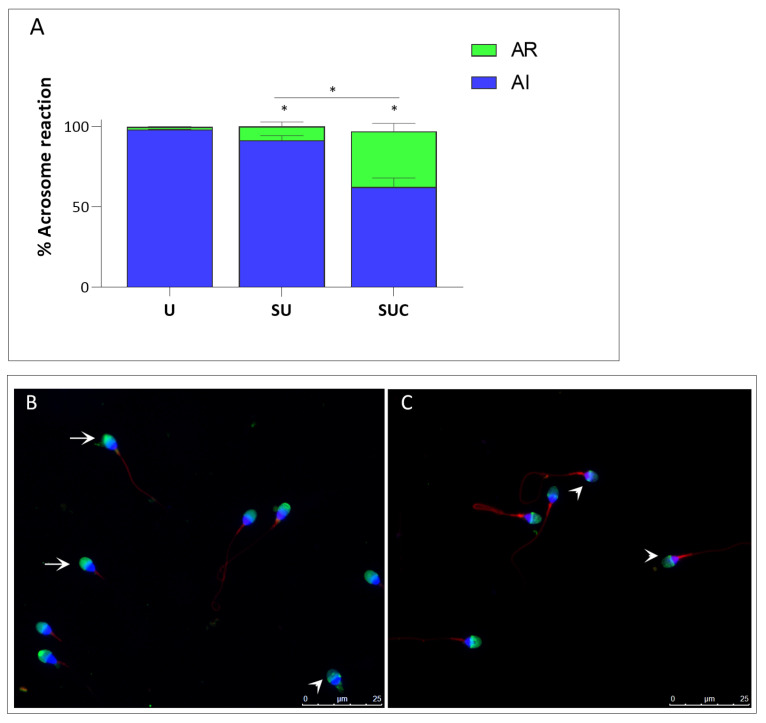
Effect of the selection procedure on acrosome status. (**A**) Percentage of acrosome reaction in unselected sperm (U) and in sperm selected by swim-up (SU) and swim-up combined with exposure to the secretome of CCs (SUC). A total of 10 semen samples for each condition were analyzed during the experiment. Blue bars: acrosome-unreacted cells; green bars: acrosome-reacted cells. Values are mean percentage ± SD. * Significantly different from control sperm (*p* < 0.05). (**B**,**C**) Fluorescence patterns of human spermatozoa selected by SUC (**B**) or SU (**C**) stained with PSA (green) for the assessment of acrosome status. Sperm were counterstained with DAPI (blue) to stain the nucleus. MitoTracker (red) stains the mitochondria at the tail level, according to the mitochondrial membrane potential (MMP). Arrows indicate the acrosome-unreacted cells with uniform green PSA fluorescence of the acrosome cap. Arrowheads show acrosome-reacted cells with PSA staining only in the equatorial segment (original magnification: ×1000). Bars: 25 µm.

After fluorescent staining with FITC-PSA and image acquisition, the acrosome reaction rate of the sperm was calculated by two blinded operators. Figure 2 shows the mean percentage of AR in the unselected, SU, and SUC samples. As expected, the percentage of reacted acrosome increased in both SU and SUC compared with the unselected sperm samples. In the SUC group, the percentage of reacted acrosome was 34.53% vs. 8.53% of the SU group (*p* < 0.05). 

**Figure 3:**
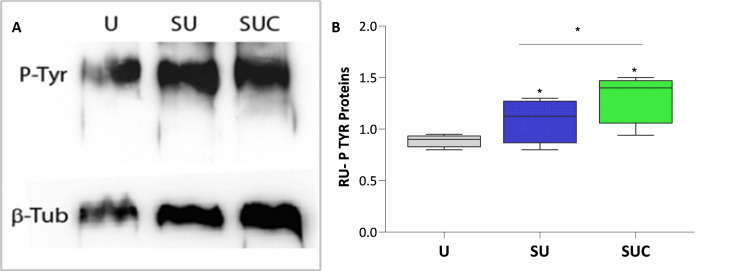
Western blot analysis of tyrosine phosphorylation level in unselected (U), swim-up (SU)-, and swim-up and CCs (SUC)-selected sperm. (**A**) Representative image of Western blot analysis of p-Tyr in the three study groups. β-tubulin was used as a loading control. The figure is representative of 3 separate experiments. (**B**) Whisker-plot graph showing the overall relative intensity of the spots as determined by computer-assisted densitometric analysis. Data show the means ± SD of relative units (RU). * *p* < 0.05.

Then, we analyzed the phosphorylation level of proteins containing tyrosine residues in both unselected and selected sperm, as the progression of capacitation is linked to the Tyr-P level [22]. To achieve this, we utilized both Western blot and immunofluorescence techniques. The Western blot comparison of tyrosine-phosphorylated protein levels between the three study groups is shown in Figure 3A; all samples show the band of about 200 kD corresponding to the family of proteins with tyrosine-phosphorylated residues. The relative quantification of the spots, shown in Figure 3B, demonstrated that SU and SUC selection procedures lead to an increase of sperm Tyr phosphorylation; this increase was about 25% in the case of SU and about 45% in the case of SUC when compared with unselected sperm (*p* < 0.05).

**Figure 4:**
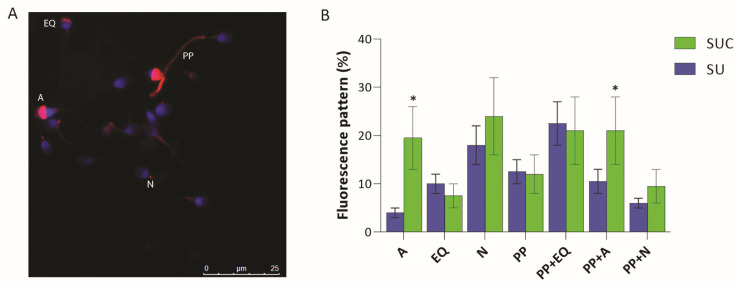
(**A**,**B**) Immunofluorescent localization of phosphotyrosine residues in human sperm. (**A**) Representative image showing phosphotyrosine staining in different sperm regions. (**B**) P-Tyr staining classified according to cellular localization in sperm selected by swim-up (SU) or swim-up combined with CC secretome exposure (SUC). Sperm were classified according to their pattern of fluorescence, and the percentage of each sperm pattern for each patient was then calculated. Data represent the mean proportion of each pattern (±SEM). In each sample, 200 sperm were evaluated. A, acrosome; EQ, equatorial region; N, neck; PP, principal piece. * *p* < 0.05.

The immunofluorescence analysis (Figure 4) confirmed the increase in Tyr phosphorylation in sperm selected with the procedure that used both swim-up and CC incubation. Indeed, in SUC-selected sperm, a larger proportion of sperm contained tyrosine-phosphorylated proteins (83%), while this proportion decreased to 75% in the SU-selected group (*p* < 0.05). Interestingly, this approach detected variations in the regional localization of phosphotyrosine in SU- versus SUC-selected sperm (Figure 4B). Phosphotyrosine residues have been identified in various compartments of spermatozoa: the acrosomal cap; the equatorial segment; the neck, and the principal piece, as well as the combined principal piece and acrosomal region, principal piece and equatorial segment, and principal piece and neck region. 

When we compared the phosphotyrosine staining in the SU and SUC samples, we observed the presence of different staining patterns occurring at different percentages. It is of interest that in the SUC-selected sperm there was a higher prevalence of sperm with tyrosine phosphorylation in the acrosomal cap and in the combined principal piece plus acrosomal region. 

### 3.3. Assessment of Mitochondrial Functionality

MitoTracker is a reliable tool for monitoring the mitochondrial membrane potential (MMP) of a sperm sample [23]. It works by staining the midpiece of a variable percentage of sperm. To determine whether the SUC procedure is more effective in isolating functional sperm than the standard swim-up technique, we evaluated the number of MitoTracker-positive sperm in each sample. The mean percentage of sperm stained with MitoTracker (assessed by fluorescence microscopy; Supplementary Figure S1) was higher for the SUC group and lower for the SU samples (86% and 76%, respectively, OR: 0.53, CI: 0.31–0.90, *p* < 0.01). 

**Figure 5:**
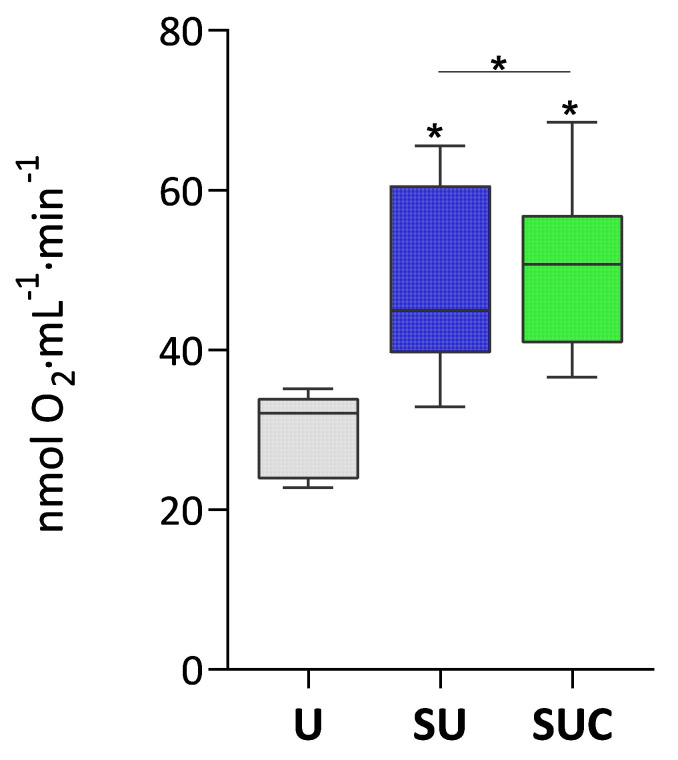
Oxygen consumption, expressed as nmol O_2_·mL^−1^·min^−1^, of unselected sperm (U) and swim-up (SU)- and swim-up combined with CCs (SUC)-selected sperm. Graphical diagrams are plotted as box–whisker plots, where boxes show the interquartile range with median and mean values, and whiskers represent the min and max confidence intervals. * *p* < 0.05. A total of four semen samples were analyzed during the experiment.

To further confirm the effectiveness of the SUC procedure in isolating high-functional sperm, we analyzed their oxygen consumption by using oxygraphic analysis; this serves as an index of oxidative phosphorylation and, consequently, ATP production. As shown in Figure 5, the SU procedure causes a significant increase in oxygen consumption of 60% (*p* < 0.01), whereas the modified SUC procedure assures an increase of up to 75% in oxygen consumption when compared with the unselected sperm samples (*p* < 0.01).

### 3.4. DNA Fragmentation in Selected Sperm

**Figure 6:**
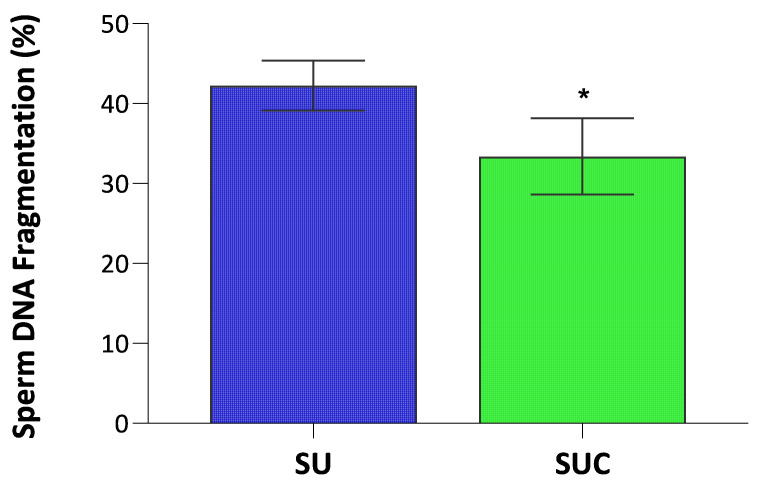
Sperm DNA fragmentation in sperm selected by swim-up (SU) and swim-up combined with CC exposure (SUC). * *p* < 0.05. A total of ten semen samples were analyzed during the experiment.

To assess the effect of selection methods on sperm DNA fragmentation (SDF), we compared the SDF of swim-up-only-prepared sperm with the CC and swim-up combined selection by using the Halo sperm procedure. The mean SDF in SU-selected sperm was 42% (s.d.: 15%, range: 28–57%); the exposure to the CC secretome allows the selection of sperm with a significantly lower SDF (33%, s.d.: 11%, range: 20–45%; *p* < 0.05) (Figure 6). Therefore, this provides further evidence that the SUC procedure is able to select sperm with lower DNA damage.

## 4. Discussion

The present study shows that the sperm selection procedure taking advantage of the cumulus cell secretome allow recovering sperm of higher quality in terms of acrosome reaction, DNA integrity, and mitochondrial functionality. Therefore, our results suggest that SUC is a good method to select sperm in the IVF laboratory. 

Our study demonstrated that the interaction of sperm with the CC secretome that occurred when using the SUC procedure allows isolating sperm with a higher percentage of reacted acrosomes. This is consistent with other studies in the literature, showing that sperm that penetrated through the cumulus oophorus had higher percentages of reacted acrosomes [11,12,24]. It has been reported that the pre-ovulatory human cumulus oophorus and mural granulosa cells were associated with activity capable of initiating the human sperm acrosome reaction in vitro [25]. Stock and colleagues [26] have demonstrated that a co-culture of spermatozoa with COC increased the percentage of acrosome-reacted spermatozoa from 15% to 31% when compared with spermatozoa not incubated with COC. The mechanism responsible for this activity of cumulus cells is still under debate. Some studies have pointed to progesterone as an important factor that can induce the acrosome reaction (Meizel et al., 1997 [25]), while others have indicated that the hyaluronic acid in the extracellular matrix of COC plays a critical role in this process [12].

Based on our findings related to the tyrosine phosphorylation footprint, we confirmed that the cumulus cell secretome is effective in promoting sperm capacitation. Indeed, the level of P-Tyr was increased by about 45% in sperm selected by the SUC method, while an increase of only 25% was detectable in sperm selected by standard swim-up. The increased proportion of sperm showing tyrosine phosphorylation staining is consistent with the acquisition of hyperactivated motility, which is required for zona pellucida penetration [27]. Notably, we detected that sperm exposed to the CC secretome showed a higher level of P-Tyr in the acrosome. It has been reported that the phosphorylation of tyrosine residues in the head of sperm is a subsurface event occurring early during capacitation and is closely related to the acquisition of the ability to display P-stimulated ARs [28,29].

Sperm function and quality are closely linked to proper mitochondrial activity [30]. In humans, the mitochondrial membrane potential (MMP) is a valid indicator of mitochondrial functions. MMP has been found to be associated with sperm viability [31] and the ability to undergo acrosome reaction [32] as well as the capacity to fertilize [33]. In fact, it has been reported that sperm with high MMP levels are generally more competent [23]. In the present study, MMP was characterized by staining with MitoTracker, whose accumulation is MMP-dependent [34].

When sperm selection was accomplished by using swim-up associated with CC exposure, the mean percentage of MitoTracker-stained sperm was higher compared with conventional swim-up. This leads us to hypothesize that the interaction with the molecules secreted by cumulus cells allows the recovery of sperm with better motility and higher fertilization capability. This is confirmed by the oxygraphic analysis, showing a significant increase in oxygen consumption in sperm recovered by the SUC method. This implies an increased production of ATP, which is positively correlated with sperm motility. Indeed, Ruiz-Pesini and colleagues reported that the mitochondrial membrane potential and the Oxygen Consumption Rate (OCR) are positively associated with ATP content, the proportion of motile sperm, and sperm velocity [35]. Therefore, we propose that exposure to the CC secretome during swim-up enhances the selection of sperm with better mitochondrial functionality, both in terms of MMP and OCR.

Conventional swim-up and density gradient centrifugation are gold-standard techniques for sperm preparation in IVF laboratories; however, some studies pointed to an increase in the DNA fragmentation level in selected semen samples, particularly in those with poor sperm parameters, probably due to the excessive amounts of ROS produced during manipulation and centrifugation [36,37,38,39]. In the present study, sperm DNA fragmentation was significantly lower in sperm selected by swim-up combined with CC exposure than in the swim-up-only group; this leads us to hypothesize that CCs may mitigate the effect of ROS. Our findings are in agreement with a study reporting a decline in the percentage of A3-positive sperm (an indicator for abnormal chromatin packaging) in COC-penetrated spermatozoa compared with control spermatozoa [24]. This is not surprising, since there is evidence that cumulus cells undergo metabolic events to reduce oxidative stress in fertilization media [6,40].

## 5. Conclusions

The exposure of sperm to CC secretome during swim-up selection assures recovery of better male gametes. This activity of cumulus cells is mediated via the secretory products of these cells. This interaction allows improved acrosome reaction, sperm capacitation, and mitochondrial activity. Despite the main limitations of this study, such as the limited sample size, our data suggest SUC as a more physiological and integrated method of sperm selection. Therefore, optimizing sperm preparation before IVF to improve sperm capacitation and minimize the DNA fragmentation effect would definitely be beneficial for ART outcomes.

## Data Availability

The raw data supporting the conclusions of this article will be made available by the authors without undue reservation.

## Supplementary Materials

The following supporting information can be downloaded at: https://www.mdpi.com/article/10.3390/cells12192349/s1, Supplementary Table S1: List of antibodies used in this study; Supplementary Table S2: List of patients’ (*n* = 27) characteristics and seminal values; Supplementary Figure S1: MitoTracker staining of SU- and SUC-selected sperm * *p* < 0.05.
