# Supplementary material for: Exposure to Cumulus Cell Secretome Improves Sperm Function: New Perspectives for Sperm Selection In Vitro
Source: Cells. 2023 Sep 25;12(19):2349. doi: 10.3390/cells12192349 (PMC10571658; doi:10.3390/cells12192349)
Supplement: Supplementary file 1 [file cells-12-02349-s001.zip › Supplementary Materials Table.docx]

**Supplementary Materials**

**SupplementaryTable S1.** List of antibodies used in this study.

| Antigen | Donor Species | Dilution | | Manufacturer | RRID |
| --- | --- | --- | --- | --- | --- |
|  |  | IF | WB |  |  |
| ***Primary antibodies*** | | | | | |
| P-Tyr | Mouse | 1:50 | 1:250 | Santa Cruz Biotechnology | AB_628123 |
| Tubulin | Mouse | - | 1:1000 | Sigma Aldrich | AB_628123 |
| ***Secondary antibodies*** | | | | | |
| Anti-Mouse IgM FITC | Goat | 1:400 | - | Sigma Life Science | AB_259799 |
| Anti-Mouse-IgG HRP | Goat | - | 1:2000 | Bio-Rad Laboratories | AB_609692 |

**Supplementary Table S2.** List of patients’ (n = 27) characteristics and seminal values.

| **Parameters** | **Average** | **Range** |
| --- | --- | --- |
| Age (years) | 34.2 | 16–51 |
| BMI | 23.5 | 17.63–32 |
| Seminal volume (mL) | 3.1 | 1.5–7 |
| Number of spermatozoa (×10^6^)/mL | 75.4 | 20–240 |
| Total number of spermatozoa (×10^6^) | 208.3 | 57.6–455 |
| Motility (%) | 65 | 47–78 |
| Morphologically normal sperm (%) | 9 | 4–16 |
