# Supplementary figures and images for: Exposure to Cumulus Cell Secretome Improves Sperm Function: New Perspectives for Sperm Selection In Vitro
Source: Cells. 2023 Sep 25;12(19):2349. doi: 10.3390/cells12192349 (PMC10571658; doi:10.3390/cells12192349)

**Supplementary Materials**


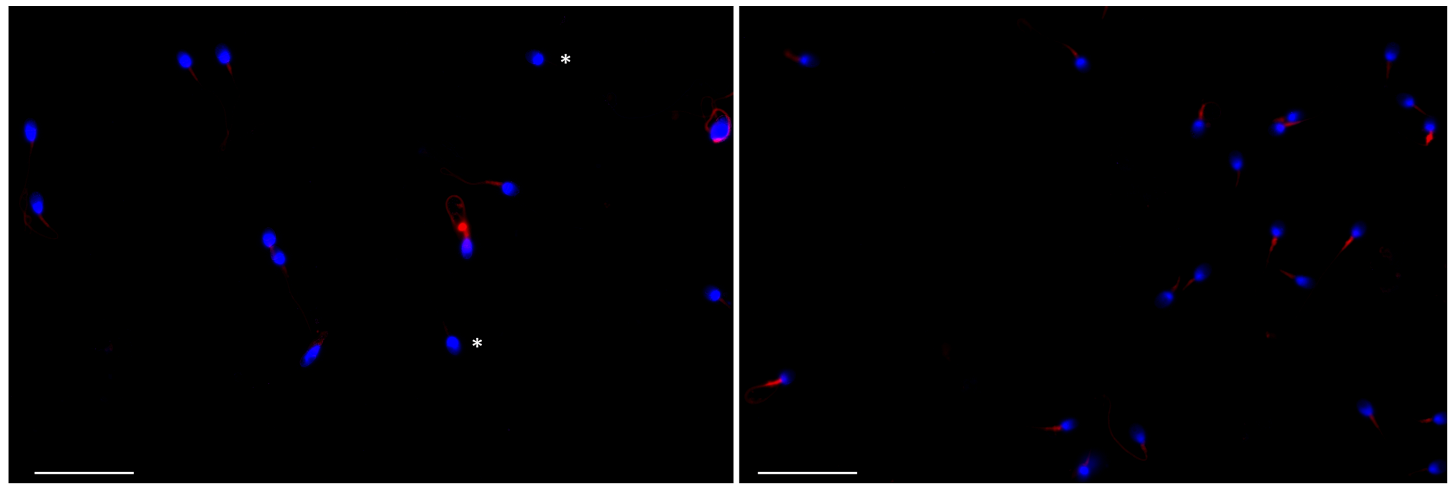


**Figure S1.** MitoTracker staining of SU- and SUC-selected sperm * *p* < 0.05.

Supplement: Supplementary file 1 [file cells-12-02349-s001.zip › Supplementary Materials Figure.docx]
